# Evidence for Brassinosteroid-Mediated PAT During Germination of *Spathoglottis plicata* (Orchidaceae)

**DOI:** 10.3389/fpls.2018.01215

**Published:** 2018-08-17

**Authors:** Stacey Novak, Nataly Kalbakji, Kylie Upthegrove, Wesley Neher, Jay Jones, Jazmin de León

**Affiliations:** ^1^Department of Biology, University of La Verne, La Verne, CA, United States; ^2^Southern California College of Optometry at Marshall B. Ketchum, Fullerton, CA, United States; ^3^Department of Botany and Plant Sciences, University of California, Riverside, Riverside, CA, United States; ^4^Ross University School of Veterinary Medicine, Saint Kitts, West Indies

**Keywords:** polar auxin transport, brassinosteroids, protocorm hairs, protocorm elongation, orchid, actin bundling, *Spathoglottis plicata*

## Abstract

Polar auxin transport (PAT) is facilitated by polar localization of PIN-FORMED (PIN) efflux carriers, which direct auxin flow and regulate developmental events. Brassinosteroids (BRs) and auxin work synergistically to promote growth, and in root geotropisms this cross-talk involves BR-directed polarization of PIN through the mobilization of F-actin. However, the role of BR in PAT during shoot growth, hair formation, and embryogenesis has not been well studied. Orchid seed are mature at a point in development that is analogous to the globular-stage of embryogenesis in typical angiosperms. Thus, this system provided a unique opportunity to study the effects of BR on PAT during embryogenesis-like events, including meristem/first leaf formation and protocorm/stem development, which is followed by protocorm hair formation. In this work, the degree to which BRs rescued embryo-like protocorms from the impact of PAT-disrupting agents, such as PAT inhibitors or high auxin levels, was determined based on growth responses. This study first established that auxin and BRs work together synergistically to promote seedling elongation in *Spathoglottis*. Repressed seedling growth caused by the PAT-disrupting agents was alleviated with eBL, suggesting that BRs enhance PAT in embryogenesis-like stages of young protocorms. However, similar responses were not evident in seed embryos. Results from this study also suggested that BRs may enhance orchid protocorm elongation by regulating auxin transport through an F-actin-mediated mechanism. With regard to protocorm hairs, increased eBL levels inhibited formation, whereas reduced BR biosynthesis altered hair patterning, and prevented outgrowth of auxin-stimulated hairs. Moreover, PAT inhibitors and repression of BR biosynthesis caused hair bud formation without hair outgrowth, suggesting a role for BR in PAT during protocorm hair development.

## Introduction

Brassinosteriods (BRs) are an essential class of plant growth regulators, of which brassinolide is deemed the most bioactive. This hormone has been implicated in a number of plant processes, including cell elongation, root growth, stress tolerance, stomatal development, patterning of vascular bundles, cell cycle activity, lateral root initiation, photomorphisms, and geotropisms (Fridman and Savaldi-Goldstein, 2013). BRs exhibit cross-talk with auxin in regulating plant development, often through a synergistic relationship (Tian et al., 2018). This interdependence is evident in common gene targets for BR and auxin (Nemhauser et al., 2006; Chung et al., 2011; Walcher and Nemhauser, 2012), the induction of auxin biosynthesis by brassinolide (Chung et al., 2011; Maharjan et al., 2014), as well as a role for BRs in the directional flow of auxin through PIN-FORMED (PIN) protein (Li et al., 2005).

Auxin is a phytohormone that morphs the plant body during development and is involved in meristem and cotyledon formation during embryogenesis, as well as boundary definition and leaf initiation from the shoot apical meristem. Polar auxin transport (PAT) and the resulting auxin concentrations within tissues help to regulate these developmental transitions (Baster and Friml, 2014). Researchers have demonstrated that PIN-FORMED protein is delivered to the membrane through vesicle trafficking, and asymmetrical positioning of PIN dictates cellular export of auxin with specific directionality (Adamowski and Friml, 2015). In addition to continuous cycling of PIN through endocytosis, PIN polarity is thought to occur by clustering of the PIN protein and varying phosphorylation states of PIN with AGC kinases, PINOID (PID), PID-related AGC3, and the phosphatase PP2A, but details of the mechanism are not entirely understood (Michniewicz et al., 2007; Baluska et al., 2008; Dhonukshe et al., 2010; Huang et al., 2010; Adamowski and Friml, 2015).

The interactive role of brassinolide and auxin in plant development has been shown to impact lateral root initiation and root geotropisms, due to PAT through PIN placement. Researchers have identified a role for auxin and ROP GTPases in promoting the accumulation of fine actin, which prevents endocytosis of PIN (Lin et al., 2012; Nagawa et al., 2012). In maize coleoptiles, high auxin levels promote actin bundling (Waller et al., 2002). In rice coleoptiles and tobacco BY-2 lines, mobile actin filaments, as opposed to bundled actin, are formed in response to low auxin levels (IAA and NAA) and mediate responses through PAT and signaling (Maisch and Nick, 2007; Nick et al., 2009). Brassinosteriods have also been shown to unbundle actin and promote auxin-driven geotropic responses in roots (Lanza et al., 2012). However, reports on the BR-influenced flow of auxin in shoots only include enhanced basipetal auxin transport with brassinolide application (Li et al., 2005). There have been no studies that address the role of BRs in promoting shoot system development when growth has been repressed by abnormal PAT.

Auxins are also responsible for promotion of root hair development, which involves PAT followed by auxin accumulation and root hair-autonomous auxin signaling (Schiefelbein, 2000; Cazzonelli et al., 2013; Lee and Cho, 2013). The basipetal flow of auxin in the root tip supplies auxin to the root hair zone. This transport occurs primarily through PIN2 (Cazzonelli et al., 2013). AUX1 (an auxin influx carrier) also plays a role in accumulating auxin in the adjacent non-hair cell, which serves as a pipeline for maintaining auxin homoestasis in the developing root hair (Lee and Cho, 2013). In addition, the application of epibrassinolide has been shown to regulate root hair growth, and studies have found that BRs promote the expression of genes WEREWOLF (WER) and GLABRA2 (GL2), which impose non-hair cell fate (Kuppusamy et al., 2009; Lee and Cho, 2013). BRs also induce expression of IAA/AUX genes that inhibit auxin signaling in root hair growth (Kim et al., 2006). However, it is not clear if the lack of hair formation in response to BRs may also be connected to auxin transport.

Polar auxin transport can be disrupted with high levels of exogenously applied auxin, in addition to several types of chemical inhibitors, including Brefeldin A (BFA), 2,3,5-Triiodobenzoic acid (TIBA), *N*-1-naphthylphthalamic acid (NPA), and monensin (MSN) (Thomson et al., 1973; Morris and Robinson, 1998; Paciorek et al., 2005). While BFA and MSN interfere with recycling of PIN endosomes to the plasma membrane, NPA, TIBA, and the auxins, NAA, IAA, and 2,4-D, depolymerize and/or bundle F-actin in root cells (Morris and Robinson, 1998; Rahman et al., 2007; Dhonukshe et al., 2008; Zhu and Geisler, 2015). This may prevent the selective uptake of PIN from the plasma membrane, resulting in the interruption of polar transport and atypical auxin distribution through tissues. Agents, such as the synthetic auxin, 2,4-D, and the PAT inhibitor, TIBA, inhibit shoot formation during germination of orchid seed (Novak and Whitehouse, 2013). Application of 2,4-D also stimulates the formation of abnormally high quantities of protocorm hairs (Novak and Whitehouse, 2013).

There have been many studies on the morphological repercussions of brassinosteroids on stems and roots, but only a few have evaluated the effect of BRs in shoot formation during embryogenesis. Studies of seed development with BR-deficient or insensitive mutations have demonstrated that BR perception or biosynthesis is necessary for normal seed size and shape (Jiang et al., 2013). Several studies have shown that somatic embryogenesis is induced by BRs (Schmidt et al., 1997; Hecht et al., 2001; Pullman et al., 2003). In addition, RNA-seq data from radish embryogenesis unveiled the up-regulation of numerous genes involved in BR and auxin signaling and biosynthesis (Zhai et al., 2016). Moreover, pharmacological studies on immature wheat embryos demonstrated that a dependence on appropriate levels of BRs or BR-signaling was necessary for meristem formation in young, globular stage embryos, but older embryos were more conducive to forming a meristem under low BR levels (Bittner et al., 2015).

Orchid seed provide an opportunity to study the impact of phytohormones on early development, since seed are naturally released from the dehiscent pod at a globular-equivalent point in development and resume embryonic-like development during early germination stages (Vinogradova and Andronova, 2002; Yeung, 2017). The histologically undefined, mature orchid seed first forms a larger mass of cells called the protocorm, which develops a first leaf at the proximal end and hairs at the distal end (Novak et al., 2008; Yeung, 2017). While in nature orchids require a symbiotic fungus for successful germination and seedling establishment, orchid seed are readily grown under tissue culture conditions, such that media can be supplemented with growth-altering agents. This system provides a means to study mechanisms associated with hormone responses at an early stage of seed development, one that is not easily captured and manipulated in other angiosperms. An additional advantage to the orchid system is the delay of primary root formation. Normally, a root pole is not established until late in germination, generally after 40–50 days, enabling one to study shoot responses, exclusively, without the complication of root development (Novak et al., 2014).

The objective to this work was to establish the growth responses of germinating orchid seed to brassinosteroids and document potential synergisms with auxin, since data of this nature has not been collected for orchid systems. Once this foundational information was obtained, the ability of brassinosteroids to promote seedling elongation by countering the effects of PAT inhibitors and high auxin levels was evaluated based upon growth responses. Potential mechanisms for BR-mediated recovery were tested using latrunculin B and EDTA. Auxin induction of protocorm hair formation in orchids also allowed documentation of the inhibitory effect of brassinosteroids and unveiled a regulatory role for this hormone in hair initiation and outgrowth. Furthermore, due to the uniquely underdeveloped seed embryo, this study provided an opportunity to gain insight into a potential role for brassinosteroids during embryogenesis.

## Materials and Methods

### Plant Materials and Culture Conditions

Mature *Spathoglottis plicata* pods were harvested from wild plants grown on Oahu, Hawaii. For each set of experiments, 10–15 green pods were surface sterilized with 95% alcohol, followed by flaming. Pods were allowed to dehisce in sterile Petri dishes, and seed were gently shaken from the pods. Approximately 20 mg of seed from a mixed population of the ten pods were aseptically transferred to each microfuge tube. Seed were wetted with 70% alcohol (v/v), briefly rinsed in demineralized water (DMW), and soaked for 1 min in 0.2% aq. NaOCl. Each group of seed was rinsed ten times in sterile DMW before being cultured onto sterile Phytamax Orchid Maintenance Media (Sigma-Aldrich Chemical, St. Louis, MO, United States) with 1% (w/v) agar at a pH of 6.5. For all experiments a total of ∼500 seed were sown on each of 125 plates to ensure adequate numbers for replicates in subsequent sub-culturing steps. Experiments were performed in batches, each requiring 24–60 plates of seed. For all experiments, seedlings were cultured in a plant growth chamber at 25°C on an 8/16 h light/dark cycle under cool white lights (Lab-Line Instruments, Melrose Park, IL, United States).

### Treatments

After 10 days, seedlings reached an early protocorm stage and were ready for sub-culture onto experimental media. Stock solutions of growth regulators, latrunculin B, PAT inhibitors, and hormone biosynthesis inhibitors were added to standard culture media after it was cooled to 50°C. For the first set of experiments, two auxins, 1-naphthaleneacetic acid (NAA) and indoleacetic acid (IAA) (Sigma-Aldrich Chemical), an auxin biosynthesis inhibitor, 4-biphenylboronic acid (BBo) (Sigma-Aldrich Chemical), epibrassinolide (eBL) (Sigma-Aldrich Chemical), and an inhibitor of brassinosteroid biosynthesis, brassinazole (BRZ) (Spectrum Chemical, Gardena, CA, United States), were added to culture media at final concentrations of 2 μM (auxins), 60 μM (BBo), 10 μM (eBL), and 100 μM (BRZ), respectively. Combination treatments were done, whereby eBL or BRZ was tested with each of the auxins and BBo. For the PAT inhibitor experiments, brefeldin A (BFA) (Sigma-Aldrich Chemical), 2,3,5-Triiodobenzoic acid (TIBA) (Sigma-Aldrich Chemical), *N*-1-naphthylphthalamic acid (NPA) (Supelco, Bellefonte, PA, United States), or monensin (MSN) (MP Biomedicals LLC, Solon, OH, United States) were added to culture media with a final concentration of 30 μM for BFA and 100 μM for the other three PAT inhibitors. Protocorm stage seedlings were cultured onto 2 μM 2,4-dichlorophenoxyacetic acid (2,4-D) (Sigma-Aldrich Chemical), or 200 μM IAA or 100 μM NAA-containing media alone or in combination with eBL. Seedlings were also cultured on three concentrations (0.5, 2, or 10 μM) of latrunculin B (Lat B) with or without eBL. All experimental treatments were conducted on four replicate plates with approximately 150-200 seedlings each. A control was included for each set of experiments. An additional control using a combination treatment of BRZ plus eBL was conducted to ensure that BRZ was not eliciting a growth-inhibiting response except through BR biosynthesis. Seedlings were grown for an additional 15 days until they reached 25 days after culture (DAC). In the case of the growth-inhibiting auxin treatments, 2,4-D, NAA or IAA, or the TIBA treatment, with and without eBL, a sub-population of seedlings cultured at 10 DAC remained in culture an additional 20 days for data collection at 45 DAC. All seedlings were fixed in 4% paraformaldehyde, and stored at 4°C until needed for data collection, unless otherwise indicated.

Some of the cultured seedlings remained in standard media until 30 days, when first leaves had formed and protocorm hairs had just begun to appear. These seedlings were sub-cultured from standard media onto four replicate treatment plates with media containing 2 μM 2,4-D, with or without 10 μM eBL, eBL alone, or 100 mM BRZ and grown for an additional 15 days (45 DAC). Other 30 day seedlings were similarly cultured onto 3 mM EDTA with or without 10 μM eBL and grown for an additional 5 days (35 DAC).

In a separate experiment, orchid seed were surface sterilized as described above and soaked in eBL (10 μM), BRZ (100 mM), eBL plus BRZ, or water overnight. Seed were then placed on culture media containing the same chemical treatment, and seedlings were cultured for 35 days under the growth promoting conditions described above.

### Microscopy

Digital images of seedlings were captured using standard and dissecting compound microscopes as well as a scanning electron microscope (SEM). For the SEM studies, paraformaldehyde-fixed orchid seedlings were dehydrated in a graded ethanol series and critical point dried using carbon dioxide in a Tousimis Samdri-795 critical point dryer. A Bio-Rad Polaron E5100 sputter coater was used to apply a fine layer of gold to the seedlings. Whole seedlings were imaged, and protocorm hair cluster data were obtained using a JEOL 6460LV SEM with an accelerating voltage of 15 kV.

### Data Collection and Statistics

For the 25 DAC seedlings, random samples of 80 seedlings were collected from the replicate plates for each treatment. Data were taken on the following growth parameters: protocorm width (PW), protocorm length (PL), first leaf length (FLL), and first leaf basal (FLB) width (**Supplementary Figure S1**). However, the high NAA and the 2,4-D treatment had low first leaf initiation and, as a result, leaf data were collected on 55 NAA-treated and 15 2,4-D-treated seedlings. Sample sizes of 40 were used for data collection on the seedlings treated with 2,4-D, TIBA, and high NAA or IAA levels and grown to 45 DAC. Data was also collected on percentage of seedlings with first leaf formation and percentage with hair or hair initiation. These data were calculated from 150 seedlings for each treatment in three groups of 50 (monensin with eBL or BRZ and BFA with BRZ caused necrosis and data collection was not possible). Of those treatments that had at least 40% of the seedlings with hairs or initiation, the percentage of seedlings in each category (hairs only, hair initiation only, or a combination of hairs and initiation) was determined for 60 seedlings, in three groups of 20. For the type of first leaf formation, abnormal/fleshy versus normal morphology, percentage data was collected on 150 seedlings, three groups of 50. For rhizome-like formation in the 2,4-D treated seedlings, percentage data were collected on three groups of 15 seedlings. SEM data were taken from thirty 45 DAC seedlings for the following parameters: number of visible protocorm hair clusters per seedling and number of hair cells per cluster. Numerical data was not collected on the 45 DAC 2,4-D/eBL or 35 DAC 2,4-D/EDTA-treated seedlings, since all of the seedlings exhibited the same response for a given treatment. NIS-Elements D imaging software version 4.12 (Nikon Instruments Inc., Melville, NY, United States) was used to determine seedling growth parameters. Data were tested for significant differences among treatments using ANOVA followed by Tukey’s HSD.

## Results

### Epibrassinolide and Auxin Synergistically Promoted Elongation in Young Protocorms, and Orchid Seed Germination Was Repressed by Epibrassinolide and Brassinazole

**FIGURE 1:**
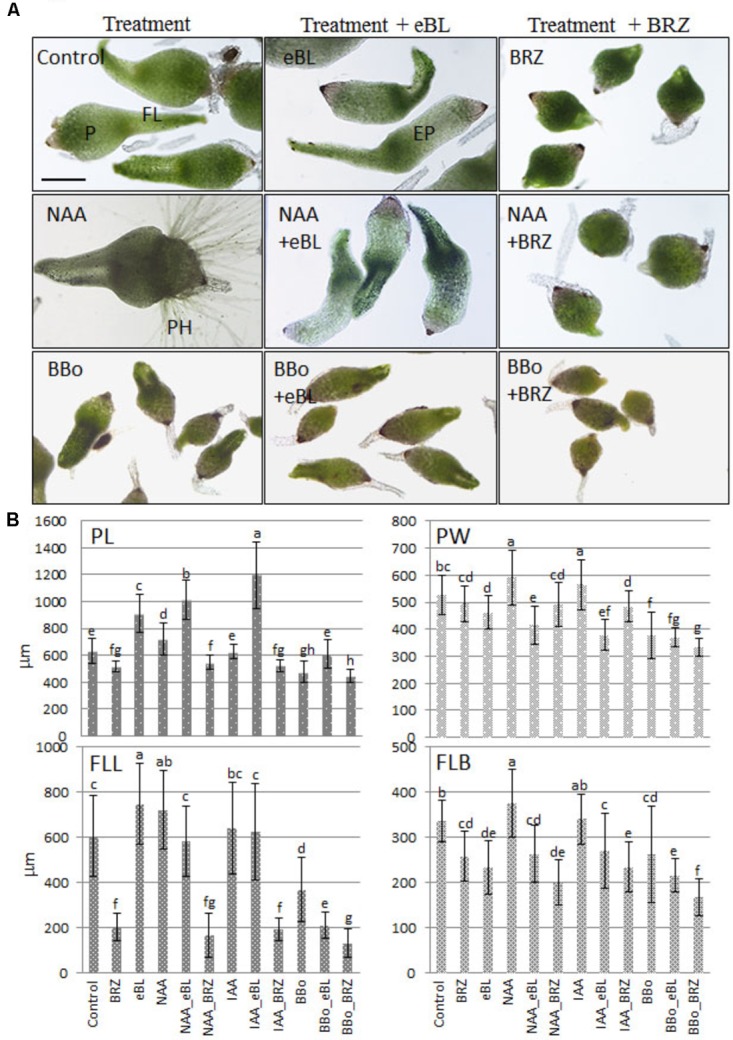
Seedling morphology **(A)** and growth parameters **(B)** of 10 DAC protocorms that were sub-cultured onto media containing hormones, hormone biosynthesis inhibitors, or combination treatments for an additional 15 days (25 DAC). eBL, epibrassinolide; BRZ, brassinazole, an inhibitor of brassinosteroid biosynthesis; NAA, 1-naphthaleneacetic acid; BBo, 4-biphenylboronic acid, an inhibitor of ABA biosynthesis; P, protocorm; EP, elongated protocorm; FL, first leaf; PH, protocorm hairs; PL, protocorm length; PW, protocorm width; FLL, first leaf length; FLB, first leaf base. The data are presented as means ± standard deviation (*n* = 80). Letters indicate shared significance groups between treatments for a given growth parameter (Tukey’s HSD) at *p* ≤ 0.05. Scale bar = 400 μm.

General growth responses of early protocorm stage seedlings to BRs and auxin were established by culturing 10 DAC protocorms for an additional 15 days with the indicated plant growth regulator and/or hormone biosynthesis inhibitor (**Figure 1**). The overall growth in response to exogenous eBL was elongated, slender seedlings, while the lack of BR biosynthesis significantly restricted growth (**Figure 1A**). Epibrassinolide alone promoted elongation of the protocorm, and to a lesser degree, the leaf, when compared to the control seedlings. Consistent with this result, inhibition of brassinosteroid biosynthesis with BRZ treatment had an inhibitory effect on protocorm and leaf development, causing severely stunted leaves and reduced protocorm widths and lengths (**Figure 1B**). Growth-promoting levels of the auxin, IAA, increased protocorm widths, while NAA enhanced growth of all measured parameters when compared to the control seedlings (**Figure 1B**). BBo, an inhibitor of auxin biosynthesis, had the opposite effect, exhibiting an overall reduction in growth of the protocorm and leaf (**Figure 1**).

Seedlings treated with a combination of auxin and eBL exhibited a notable increase in protocorm length when compared to those treated with IAA, NAA, or eBL alone (**Figure 1**). However, leaf lengths in these treatments did not increase, rather eBL caused a reduction in leaf length of NAA-treated seedlings, and the IAA-treated seedlings remained unchanged in leaf length (**Figure 1B**). Other growth parameter changes with the addition of eBL to the auxin-treated seedlings included a decrease in protocorm and leaf base widths. When auxin levels were diminished with BBo treatment, eBL improved protocorm lengths, but lengths were not comparable to eBL-treatment alone (**Figure 1B**). Auxin (IAA or NAA), in combination with the brassinosteroid biosynthesis inhibitor, BRZ, did not improve growth over the treatment with BRZ alone. Inhibiting biosynthesis of both auxin and brassinosteroids resulted in smaller protocorms with shorter first leaves than all other treated seedlings (**Figure 1**).

**FIGURE 2:**
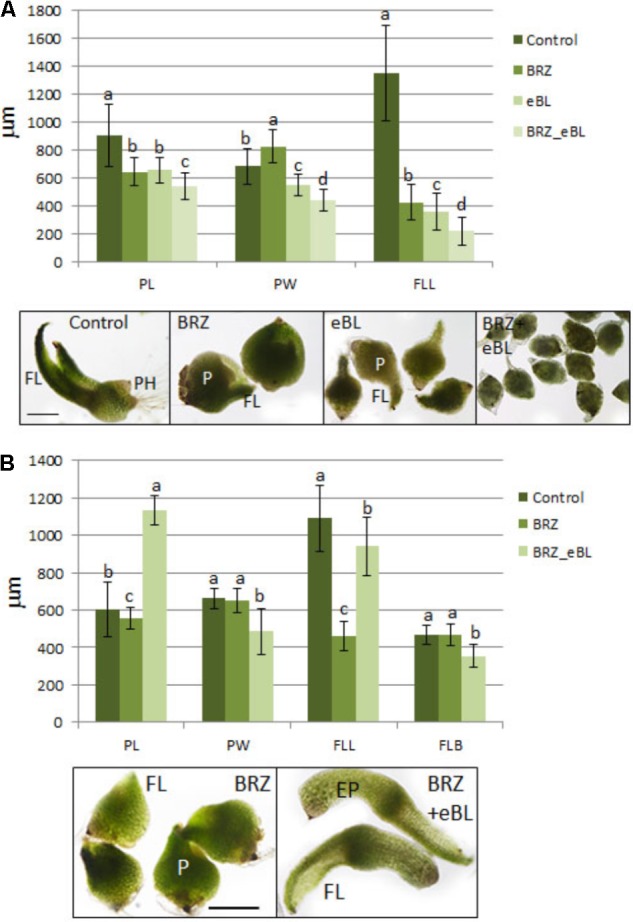
Seed embryos **(A)** and 10 DAC protocorms **(B)** cultured for 35 and 15 days, respectively, on media with epibrassinolide (eBL), brassinozole (BRZ), or a combination of these reagents. P, protocorm; EP, elongated protocorm; FL, first leaf; PH, protocorm hairs. The data are presented as means ± standard deviation (*n* = 80). Letters indicate shared significance groups between treatments for a given growth parameter (Tukey’s HSD) at *p* ≤ 0.05. Scale bar = 400 μm **(A)**. Scale bar = 500 μM **(B)**.

Seed embryos were soaked in eBL, BRZ, or a combination, and cultured with these reagents for 35 days (**Figure 2A**), and 10 DAC protocorms were sub-cultured onto the same hormone/inhibitor-containing media for an additional 15 days (**Figure 2B**). At both developmental time points, BRZ treatment produced a stunted shoot with fleshy leaves and a spherical protocorm (**Figure 2**). However, the eBL treatment yielded variable results for the two time points. Overall, eBL inhibited seed germination/shoot growth in seed embryos (**Figure 2A**), but it promoted an elongated protocorm and leaf in 10 DAC protocorms (**Figures 1A, 2B**). The eBL-treated seed embryos consistently produced a first leaf, however, they were severely stunted (**Figure 2A**). The BRZ-eBL-treated 10 DAC protocorms exhibited elongation in the leaf and protocorm that exceeded the control seedlings, confirming that eBL can reverse the effects of BRZ on 10 DAC protocorms by behaving in a manner that is functionally equivalent to endogenous BRs (**Figure 2B**). In contrast, in the seed embryo, BRZ-eBL treatment was more repressive to growth than eBL alone. Protocorms were smaller, and first leaves were less developed (**Figure 2A**).

### The Co-application of Epibrassinolide Partially Corrected Abnormal Leaf Morphology and Helped to Restore Repressed Shoot Growth Due to PAT Inhibitors or Auxin

**FIGURE 3:**
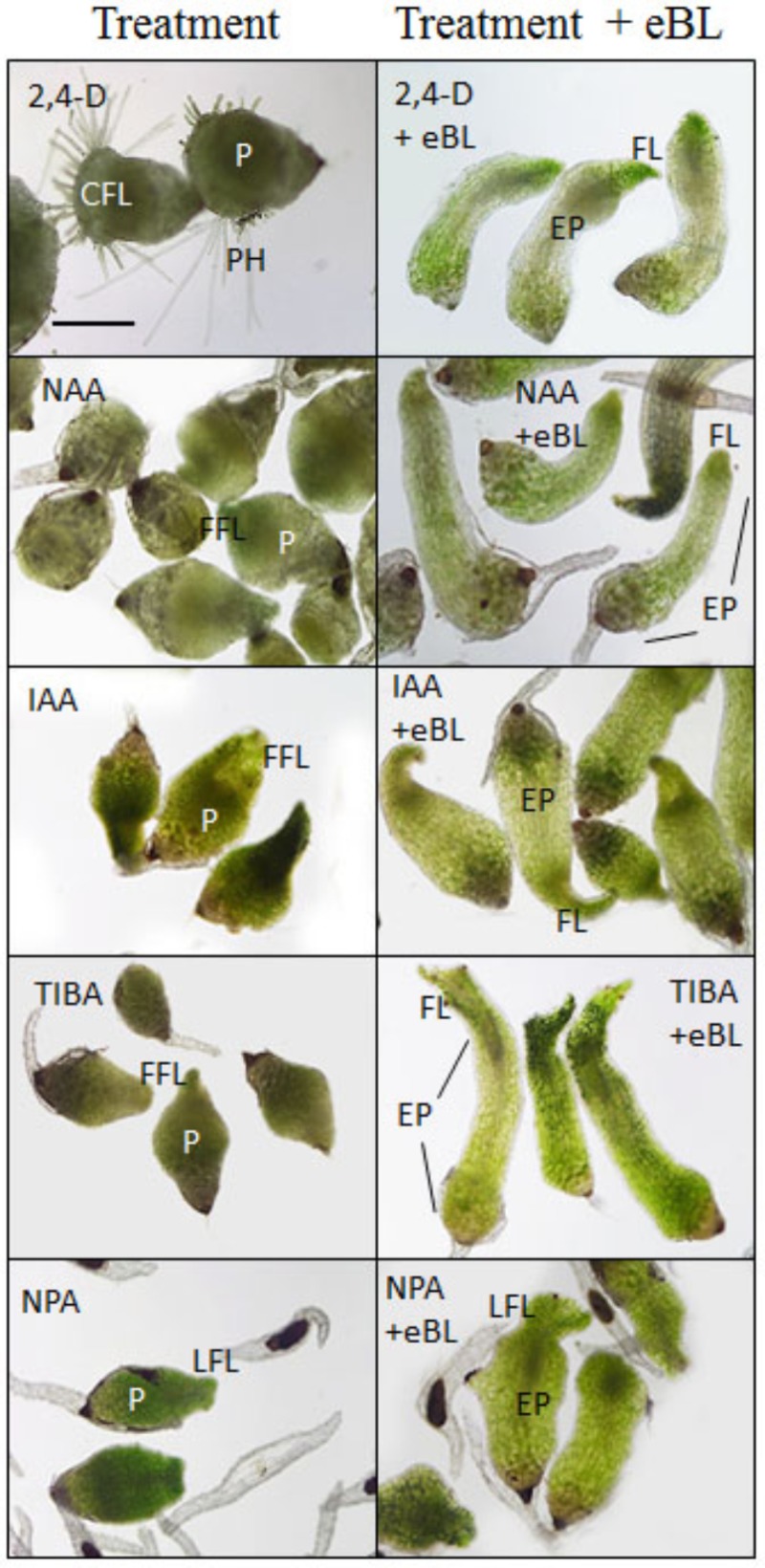
Seedling morphology for 10 DAC protocorms cultured with PAT inhibitors or growth inhibiting levels of auxin, with or without application of epibrassinolide (eBL) for an additional 15 days (25 DAC). 2,4-D, 2,4-dichlorophenoxyacetic acid; NAA, 1-naphthaleneacetic acid; IAA, indoleacetic acid; TIBA, 2,3,5-Triiodobenzoic acid; NPA, *N*-1-naphthylphthalamic acid. P, protocorm; EP, elongated protocorm; FL, first leaf; LFL, lobed first leaf; CFL, callused first leaf; FFL, fleshy first leaf; PH, protocorm hairs. Scale bar = 400 μm.

**FIGURE 4:**
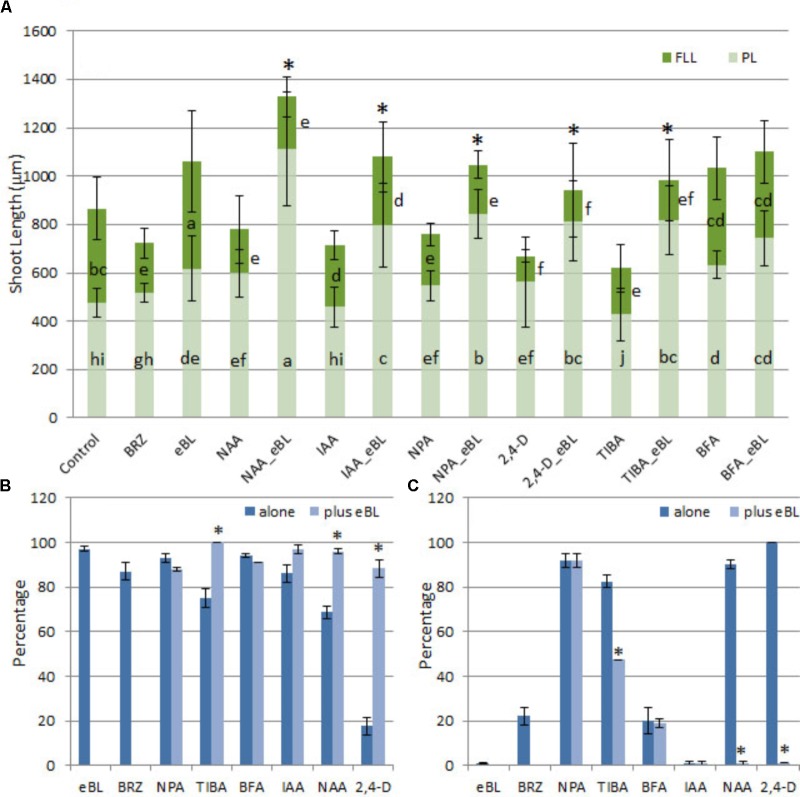
Growth parameters **(A)**, percentage of seedlings with first leaf **(B)**, and percentage of seedlings with a fleshy, callused, or lobed first leaf **(C)** for 10 DAC protocorms cultured with PAT inhibitors or growth inhibiting levels of auxin, with or without application of epibrassinolide (eBL) for an additional 15 days (25 DAC). NAA, 1-naphthaleneacetic acid; IAA, indoleacetic acid; 2,4-D, 2,4-dichlorophenoxyacetic acid; NPA, *N*-1-naphthylphthalamic acid; TIBA, 2,3,5-Triiodobenzoic acid; BFA, brefeldin A; FLL, first leaf length; PL, protocorm length. Growth parameter data are presented as means ± standard deviation (*n* = 80, except as indicated in materials and methods). Letters indicate shared significance groups for each growth parameter for different treatments (Tukey’s HSD) at *p* ≤ 0.05. Percentage data are presented as means ± standard deviation (*n* = 150, three groups of 50). Asterisks indicate a significant increase in overall shoot growth **(A)**, percentage with first leaf **(B)**, or a decrease in percentage with abnormal leaves **(C)** when eBL is added to the treatment. ^∗^Significance at *p* ≤ 0.05.

Developmental responses of young protocorms to the growth-repressing levels of auxin or PAT inhibitors were evaluated, and seedling rescue was determined for combination treatments with eBL after 25 days in culture. The PAT inhibitors (TIBA and NPA) and auxins (2,4-D, IAA, NAA) restricted growth of the seedlings to various degrees, including shortened shoots with stunted, fleshy, lobed, and/or callused leaves when compared to control seedlings; but the addition of eBL enhanced overall shoot elongation and partially restored leaf morphology (**Figure 3**). The increase in shoot growth was due to elongated protocorms in all treatments, and leaf extension was not enhanced with the addition of eBL at this 25 DAC time point (**Figure 4A**). While improved leaf morphology was evident in the combination treatments, eBL with 2,4-D, NAA, IAA, or TIBA, the NPA-eBL-treated seedlings exhibited fleshy, stunted, lobed, leaf morphology (**Figures 3, 4A**). In addition, BFA-treated seedlings did not exhibit an improvement in overall shoot growth with the addition of eBL, but there was an increase in protocorm length (**Figure 4A**). TIBA, NAA, and 2,4-D treatment caused a reduction in the percentage of seedlings with first leaf formation, but the addition of eBL to growth-inhibiting auxin levels or TIBA allowed seedlings to develop a meristem capable of generating a structure with leaf morphology (**Figures 3, 4B**). Epibrassinolide also reduced the percentage of seedlings with callused or fleshy leaves caused by TIBA, NAA or 2,4-D (**Figure 4C**).

**FIGURE 5:**
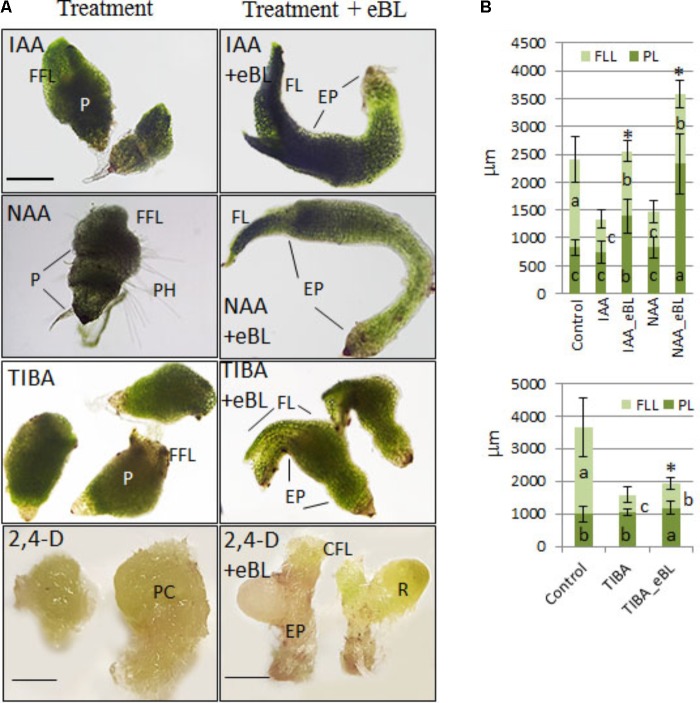
Seedling morphology **(A)** and growth parameters **(B)** of 10 DAC protocorms sub-cultured onto growth-inhibiting auxin levels or PAT inhibitors and cultured for an additional 35 days with and without epibrassinolide (eBL) (45 DAC). IAA, indoleacetic acid; NAA, 1-naphthaleneacetic acid; 2,4-D, 2,4-dichlorophenoxyacetic acid; TIBA, 2,3,5-Triiodobenzoic acid; eBL, epibrassinolide. The data are presented as means ± standard deviation (*n* = 40). Letters indicate shared significance groups between treatments for a given growth parameter (Tukey’s HSD) at *p* ≤ 0.05. P, protocorm; EP, elongated protocorm; FL, first leaf; FFL, fleshy first leaf; CFL, callused first leaf; PH, protocorm hairs; PC, protocorm/callus; FLL, first leaf length; PL, protocorm length. Asterisks indicate an increase in overall shoot growth with the addition of eBL to the auxin or TIBA treatments. ^∗^Significance at *p* ≤ 0.05. Scale bar = 2 mm for 2,4-D treatment. Scale bar = 500 μm for 2,4-D + eBL treatment. Scale bar = 400 μm for all other images.

Given an additional 20 days in culture (45 DAC), the IAA, NAA, and TIBA- treated seedlings with eBL generated very elongated protocorms when compared to those without eBL (**Figure 5**). In addition, the IAA and NAA-eBL-treated seedlings had near normal leaf morphology (**Figure 5A**), and generated leaf lengths that more closely approached those of the control seedlings than observed in the earlier time point (25 DAC) (**Figures 4, 5B**). The TIBA/eBL-treated seedlings also had improved leaf morphology and growth compared to TIBA alone, but overall seedling growth was repressed when compared to control seedlings (**Figure 5**). In contrast to results with TIBA, IAA or NAA with eBL, the 2,4-D seedling leaf morphology did not improve with additional time in culture, rather the seedlings formed callus tissue where a leaf should be located. While the 2,4-D treatment alone resulted in a large mass of callus tissue, the addition of eBL promoted the formation of smaller seedlings that had a slender protocorm from which eBL induced the development of rhizome-like structures in 95% of the seedlings (**Figure 5** and **Table 1**).

**Table 1:** Growth parameters and percentage of seedling with rhizome-like structures in 2,4-D and 2,4-D plus eBL-treated seedlings.

	PL (mm)	PW (mm)	% seedlings with rhizome-like structures
2,4-D	3.125 ± 0.845a	1.975 ± 0.697a	0
2,4-D + eBL	1.536 ± 0.211b	0.438 ± 0.048b	95.0 ± 2.0


*Letters indicate shared significance groups between treatments for a given parameter (Tukey’s HSD) at *p* ≤ 0.05.*

### Lat B Inhibition of Actin Polymerization Does Not Repress eBL-Induced Protocorm Elongation, and 2,4-D-Stimulated Protocorm Collapse Can Be Prevented With eBL or EDTA Treatment

**FIGURE 6:**
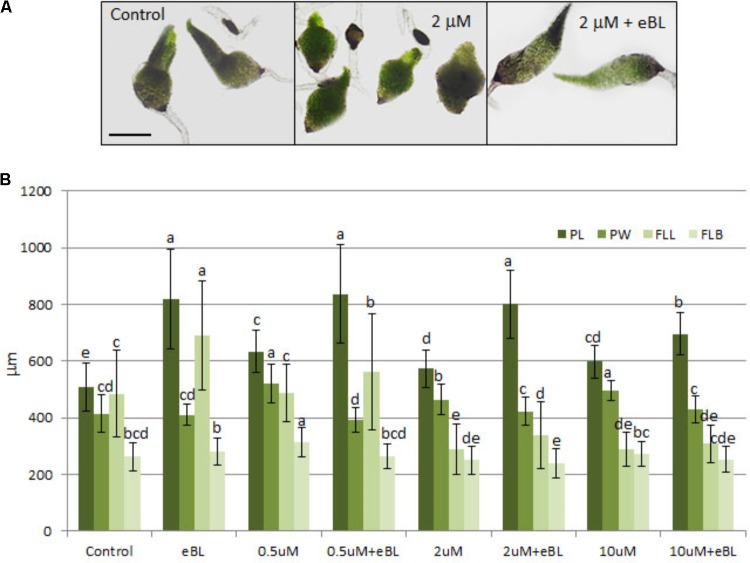
Seedling morphology **(A)** and growth parameters **(B)** of 10 DAC protocorms cultured on media containing latrunculin B (Lat B) with and without epibrassinolide (eBL) for 15 days (25 DAC). PL, protocorm length; PW, protocorm; width FLL, first leaf length; FLB, first leaf base. The data are presented as means ± standard deviation (*n* = 80). Letters indicate shared significance groups between treatments for a given growth parameter (Tukey’s HSD) at *p* ≤ 0.05. Scale bar = 450 μm.

F-actin is thought to play a role in BR-stimulated geotropisms by facilitating the polarity of auxin through assistance in PIN placement (Lanza et al., 2012; Lin et al., 2012; Nagawa et al., 2012). Since Lat B will bind monomeric actin and also promote depolymerization of F-actin (Morton et al., 2000), this drug was used to determine if F-actin formation is necessary for eBL-induced seedling elongation. Treatment with Lat B alone caused seedlings to exhibit stunted leaves and somewhat longer and wider protocorms than the control seedlings (**Figure 6A**). The combination treatment with Lat B and eBL resulted in an elongated protocorm with growth parameters similar to eBL treatment alone (**Figure 6**). First leaves were also affected by 2 and 10 μM Lat B, but there was some recovery by eBL only in the 2 μM treatment (**Figure 6**). The 10 μM Lat B plus eBL treatment became somewhat toxic to the seedlings, causing them to become partially necrotic.

**FIGURE 7:**
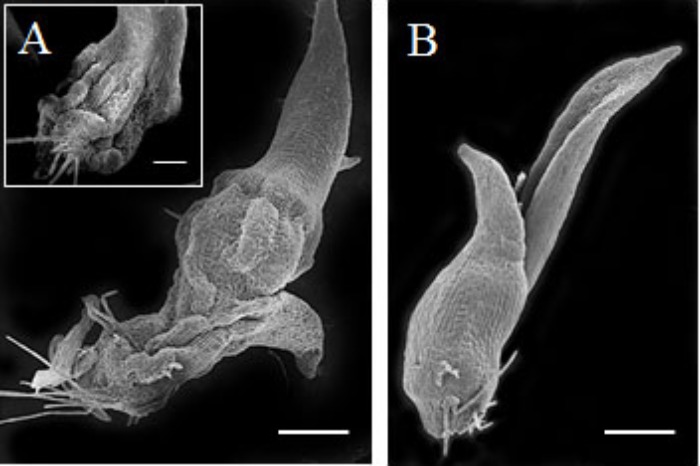
Scanning electron microscope (SEM) of 30 DAC seedlings cultured on 2,4-D **(A)** or 2,4-D plus epibrassinolide (eBL) **(B)** for 15 days (45 DAC). Inset image shows a second seedling with collapsed stem morphology. Scale bar = 500 μm. Inset scale bar = 100 μm.

**FIGURE 8:**
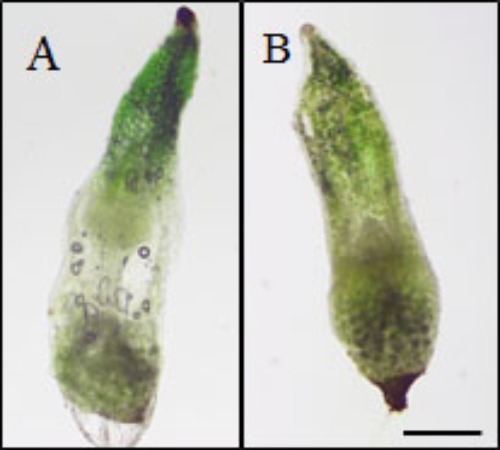
Thirty DAC seedlings cultured with 2,4-D **(A)** or 2,4-D plus 3 mM EDTA **(B)** for 5 days (35 DAC). Scale bar = 500 μm.

The mechanism by which eBL restored seedling growth in the 2,4-D seedlings was investigated by testing whether eBL recovery could be mimicked by EDTA. The addition of 2,4-D to older seedlings (30 DAC) resulted in a collapsed protocorm/stem after 5 days in culture (**Figure 7A**). This occurred in all 80 seedlings. Epibrassinolide plus 2,4-D resulted in normal protocorms/stems that lacked any evidence of collapse after 15 days in culture (**Figure 7B**). One study found that epinasty, caused by 2,4-D, resulted from the formation of free radicals that attack actin and inhibit polymerization into F-actin (Rodríguez-Serrano et al., 2014). Treatment with EDTA could prevent this response through a reduction in OH∙^-^ radical production. In this work, we tested whether seedlings could be rescued from 2,4-D induced stem collapse with EDTA treatment. After 5 days in culture, all 80 of the 2,4-D-treated seedlings showed evidence of tissue collapse in the stem (**Figure 8A**), but those treated with 3 mM EDTA and 2,4-D had no evidence of collapse (**Figure 8B**). EDTA proved toxic to the seedlings at levels higher than 3 mM, and had no protective effect at 10 μM, 100 μM or 1 mM, thus useful data could not be collected. Young protocorms were also cultured with EDTA plus 2,4-D, but no protective EDTA level could be determined. At low EDTA levels, 2,4-D caused its typical response described above, and at the higher levels, the protocorm died within 5–7 days and data collection was not possible.

### Epibrassinolide Inhibited Protocorm Hair Formation, and Regulated Patterns of Hair Initiation. Treatment With BRZ or PAT Inhibitors Promoted Hair Initiation Rather Than Hair Outgrowth

**FIGURE 9:**
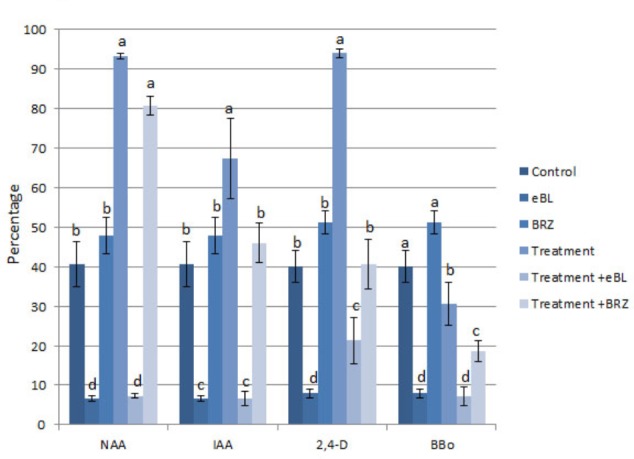
Percentage of seedlings with hairs or hair initiation after 10 DAC protocorms were cultured on auxin for 15 days, with or without epibrassinolide (eBL) or brassinazole (BRZ) (25 DAC). IAA, indoleacetic acid; NAA, 1-naphthaleneacetic acid; 2,4-D, 2,4-dichlorophenoxyacetic acid; BBo, 4-biphenylboronic acid. Letters indicate shared significance groups between treatments for a given growth parameter (Tukey’s HSD) at *p* ≤ 0.05.

**FIGURE 10:**
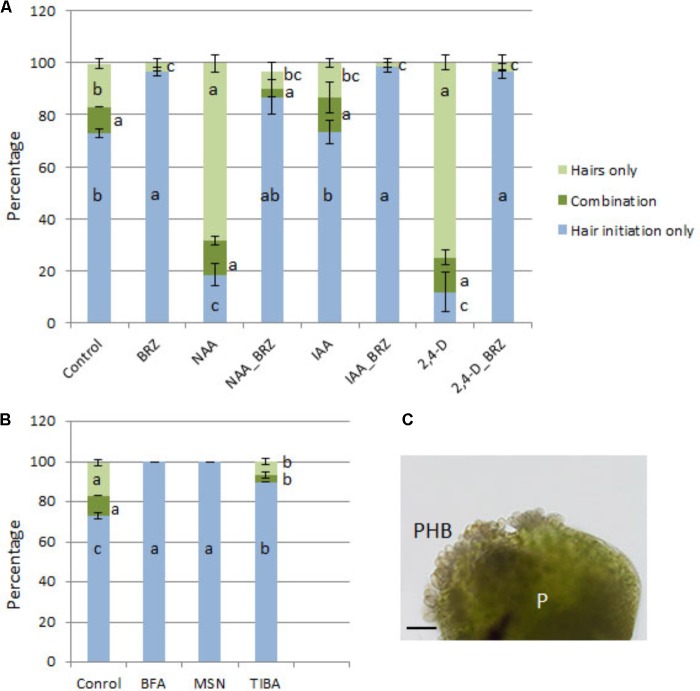
Percentage of seedlings in each of the categories: hairs only; a combination of hairs and hair initiation; and hair initiation only, after 10 DAC protocorms were cultured on auxin **(A)** or PAT inhibitors **(B)** for 15 days with or without brassinazole (BRZ) (25 DAC). High quantities of protocorm hair buds (PHB) formed in response to BRZ and NAA **(C)**. IAA, indoleacetic acid; NAA, 1-naphthaleneacetic acid; 2,4-D, 2,4-dichlorophenoxyacetic acid; BFA, brefeldin A; MSN, monensin; TIBA, 2,3,5-Triiodobenzoic acid; P, protocorm. Letters indicate shared significance groups between treatments for a given growth parameter (Tukey’s HSD) at *p* ≤ 0.05. Scale bar = 100 μm.

The percentage of seedlings with protocorm hair formation/initiation was higher in the NAA, IAA, and 2,4-D-treated seedlings than control seedlings, and combination treatments with eBL resulted in a large reduction in the percentage of seedlings with hairs (**Figure 9**). The auxin biosynthesis inhibitor, BBo, also caused a decrease in the percentage of seedlings with hairs. The BRZ-NAA-treatment resulted in a higher percentage of seedlings with hairs than the control or BRZ alone. Other auxin treatments with BRZ yielded percentages that were comparable to the control, and the BBo treatment with BRZ generated fewer seedlings with hairs than the control or BRZ alone (**Figure 9**). In treatments which produced at least 40% of seedlings with hairs or hair initiation, seedlings were further categorized into one of three following groups: (1) percentage of seedlings with hairs only (2) percentage of seedlings with hair initiation only [as defined by Schiefelbein (2000)] and (3) percentage of seedlings with a combination of hair initiation and hairs (**Figures 10A,B**). BRZ caused more hair initiation than the control. Auxin treatments, NAA and 2,4-D, resulted in most seedlings possessing hairs only, but the BRZ plus auxin-treatment generated seedlings with nearly all hair initiation (**Figure 10A**). A similar breakdown for PAT inhibitor-treatments, BFA, MSN, and TIBA, also showed that these treatments promoted hair initiation, but elongation was inhibited (**Figure 10B**). Therefore BRZ, PAT inhibitors, and auxins plus BRZ - treated seedlings had protocorms riddled with hair buds (sites of hair initiation). This was especially evident in the NAA-BRZ-treated seedlings (**Figure 10C**).

**FIGURE 11:**
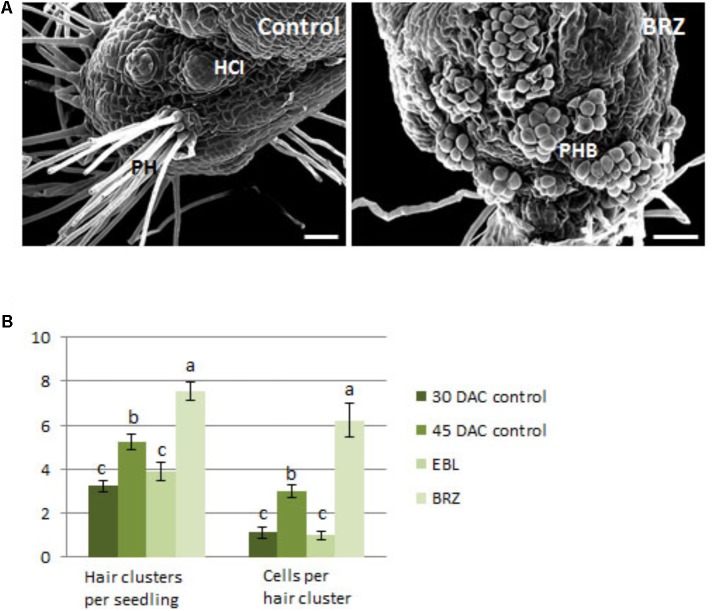
Scanning electron microscopy of the protocorm region of 30 DAC seedlings cultured for 15 days on brassinazole (BRZ) **(A)**, and counts for hair cluster number and hairs per cluster of epibrassinolide (eBL) and BRZ- treated seedlings **(B)**. PH, protocorm hairs; HCI, hair cluster initiation sites; PHB, protocorm hair buds. Letters indicate shared significance groups between treatments for a given parameter (Tukey’s HSD) at *p* ≤ 0.05. Scale bar = 100 μm.

A separate group of seedlings were sub-cultured at 30 DAC, when protocorm hairs were just beginning to form, and grown for an additional 15 days with eBL or BRZ. Scanning electron micrographs of the protocorm/stem region of these seedlings revealed a surface populated with more hair buds in large clusters in the BRZ-treated seedlings than control seedlings (**Figure 11A**). In contrast, the control seedlings had hair tufts with complete hair outgrowth. Epibrassinolide repressed hair cluster number and cells per cluster to numbers comparable to those at 30 days, the time point when seedlings were sub-cultured (**Figure 11B**). BRZ resulted in seedlings with a higher number of hair clusters and cells per cluster compared to the 45 DAC control seedlings (**Figure 11B**).

## Discussion

Brassinosteroids (BRs) enhance auxin responses, such as stem elongation, but at the same time, BRs antagonize auxin-promoted root hair formation (Nemhauser et al., 2004; Cheng et al., 2014). A common feature of these developmental events is their dependence upon PAT (Jensen et al., 1998; Jenik and Barton, 2005; Velasquez et al., 2016). Thus a focus of this work was to evaluate the role of BR in PAT. Orchid protocorm development provides a unique opportunity to study this hormone cross-talk, since PAT inhibitors suppress shoot formation and high auxin levels enhance hair formation (Novak and Whitehouse, 2013). For this work, an *in vitro* system was used to analyze the impact of BRs and their interaction with auxin through PAT during early stages of orchid development, when the protocorm, shoot meristem, and protocorm hairs were being formed.

### Impact of Epibrassinolide and Auxin on Orchid Seedling Development

In Arabidopsis, auxin and BRs work together to elicit root geotropisms (Li et al., 2005) and stem elongation (Nemhauser et al., 2004). Consistent with this work, orchid seedlings also exhibited increased protocorm elongation with the dual treatment of auxin and eBL. The morphological nature of the protocorm has not been well defined, yet it has been suggested that they are rhizome-like (Chang et al., 2005), and in this case, may be behaving like a stem in their response to the additive effect of these two hormones. In general, eBL also caused the protocorm and leaf base to slenderize, when compared to control seedlings, auxin treatments, or auxin plus eBL, suggesting that BR-regulated protocorm diameter can influence meristem and leaf morphology.

While leaf elongation was not enhanced by the combination treatment of eBL and auxin, it should be noted that inhibition of BR biosynthesis severely stunted the seedling shoots, including the leaves, and exogenously applied auxin did not rescue this condition. Consistent with this finding, eBL application alone promoted leaf elongation, similar to what has been reported in Arabidopsis (Nakaya et al., 2002). The overall reduced growth caused by diminished auxin biosynthesis (BBo treatment), was not rescued with the addition of eBL to the culture media, providing additional evidence for the requirement of both, and perhaps an interdependence of these hormones in eliciting optimal growth in orchid systems. Moreover, these results make clear the need for adequate auxin levels to be present in the tissues in order for eBL to exert its full, growth-promoting effects on protocorm elongation.

### Role of Brassinosteroids in Early Orchid Germination

*Spathoglottis plicata* embryos undergo development until a late globular-like stage at which time they are naturally released from the parental seedpod (Sriyot et al., 2015). Thus orchid seed germination provides a unique opportunity to study pseudo-embryonic time points under controlled culture conditions. These developmental stages are comparable to mid and late embryogenesis of orthodox angiosperms, a time when meristems and primordial organs take form and generate a well-defined embryonic axis. In this study, the effects of eBL and BRZ application were analyzed at two time points: mature, globular-staged seed embryos and 10 DAC nascent protocorms, which had increased in size, but had no apparent meristem. The normal pathway for auxin flow during embryo development is basipetal then acropetal to help establish the meristem and to initiate seed leaves (Jenik and Barton, 2005), after which, net flow in the stem is basipetal, promoting hypocotyl elongation and supplying auxin to the root. In this study, regardless of the time point tested, reduced BR levels (BRZ treatment) seemed to hinder auxin progression, resulting in an enlarged, bulbous, protocorm with highly stunted, fleshy leaves (note that in orchids, the root pole has not yet formed). The fleshy leaves and enlarged protocorm in the BRZ-treated seedlings were consistent with the effects of excess auxin and PAT inhibitor treatment in this study and in the study by Novak and Whitehouse (2013), thus auxin “pooling” due to reduced PAT may be the reason for this BRZ-induced seedling morphology.

There were distinctively different responses between the two developmental time points when eBL was combined with the BRZ treatment. The young protocorms regained their elongated morphology, both protocorm and leaf, while in the globular-staged seed embryos, development was significantly delayed and seedlings only reached an early protocorm/first leaf stage after 35 days of culture. Moreover, in the seed embryo, eBL alone allowed more growth than eBL/BRZ treatment, suggesting that endogenous BRs may be needed for early development. The eBL and eBL/BRZ growth differences in the two developmental time points were consistent with work done by Bittner et al. (2015), in which the authors demonstrated that early globular wheat embryos were more sensitive to appropriate BR levels than older embryos. The fact that a meristem formed regardless of time point and treatment (excess or reduced BR) suggests that BR has played its role in meristem formation prior to seed maturity in this orchid species and/or meristem establishment may be delayed but; but cannot be prevented by anomalous BR levels.

### Shoot Growth Responses to PAT Inhibitors or High Auxin Levels in Combination With Epibrassinolide

High auxin levels and PAT inhibitors disrupt polar auxin flow and have negative effects on growth (Waller et al., 2002; Paciorek et al., 2005; Novak and Whitehouse, 2013). In this study, treatment of 10 DAC protocorms with high auxin (NAA, 2,4-D or IAA) levels or PAT-inhibitors (NPA, BFA, and TIBA) resulted in repressed shoot growth, and after an additional 15 days in culture, produced seedlings with stunted, fleshy first leaves. Combination treatments with eBL resulted in increased protocorm lengths for all treatments and partially rescued first leaf morphology in the 2,4-D, IAA, NAA and TIBA-treated seedlings; but leaves remained stunted. After an additional 20 days in culture, leaf morphology and overall growth significantly improved in the eBL combination treatments with IAA, NAA and TIBA. As stated above, this study established that both auxin and BR must be synthesized for orchid seedling shoot elongation to occur (see BBo and BRZ data), and, based upon the PAT inhibitor data, auxin flow must be permitted for seedling elongation and leaf formation. Since eBL was able to provide growth recovery for germinating protocorms treated with PAT disrupting agents, BR is likely involved in facilitating auxin flow during germination of young protocorms. Similarly, one study demonstrated that the application of BRs enhanced the basipetal flow of auxin in Arabidopsis stems (Li et al., 2005). It should also be noted that the 2,4-D/eBL-treated seedlings that were given an additional 20 days in culture developed callused leaves, but, unlike 2,4-D-treatement alone, the eBL seedlings also formed rhizome-like structures. Older (35 days) orchid seedlings treated with 2,4-D also formed rhizome-like structures (Novak and Whitehouse, 2013). Thus eBL may be causing a redirection of auxin that allowed for more seedling-like morphology through the initiation of these rhizome-like structures, while 2,4-D alone only promoted callus formation.

Epibrassinolide failed to rescue leaf morphology in the NPA and BFA-treated seedlings. BFA binds to ARF-GEFs, such as GNOM, and prevents endosome recycling, which may interfere with PIN delivery, a condition that BRs cannot remedy and may be more germane to leaf development than protocorm elongation (Paciorek et al., 2005). NPA binds to a number of targets, including TWD1, ABCB (auxin transporters), and ABP1 (auxin binding protein). NPA’s inhibition of auxin responses, interruption of auxin transport, and/or inhibition of actin polymerization may be the cause of abnormal leaf morphology.

### Potential Mechanisms for BR-Regulated Protocorm Elongation

The auxin:BR synergism in root geotropisms involves the placement of PIN proteins through a means requiring free actin filaments (Lanza et al., 2012; Lin et al., 2012; Saini et al., 2015). The PAT-disrupting agents selected for this study also bundle actin (Waller et al., 2002; Zhu and Geisler, 2015), and BRs have been shown to unbundle actin in root cells (Lanza et al., 2012). Thus this study provided evidence that BR-regulated protocorm elongation in orchid seedlings may be controlled by the unbundling of actin to promote PAT. However in this work, protocorm treatment with Lat B, which prevents polymerization of actin (Morton et al., 2000), can depolymerize actin filaments (Spector et al., 1983), and bundle actin (Nagawa et al., 2012), demonstrated that new actin filament formation was not required for eBL-induced protocorm elongation. In addition, researchers have found that both auxin and BRs promote transverse microtubule orientation, which enhances hypocotyl elongation in seedlings (Sakoda et al., 1992; Catterou et al., 2001; Liu et al., 2018). Thus, it remains very plausible that auxin-enhanced, BR- promoted protocorm elongation may be a function of both actin and microtubule action.

Brassinosteroids have been shown to increase the expression of antioxidant genes, such as SOD and catalase (Bajguz and Hayat, 2009). While there have been no reports that NAA, IAA or PAT inhibitors promote free radicals, 2,4-D has been implicated in the formation of H_2_O_2_ and O_2_∙^-^ (Rodríguez-Serrano et al., 2014). In this work the protocorm/stem collapse in 30 DAC seedlings due to 2,4-D was prevented when seedlings were cultured on media that included eBL with the 2,4-D treatment. We speculated that this result may have been due to enhanced free radical protection as a result of BR-induced gene expression. This hypothesis was supported when addition of 3 mM EDTA to the 2,4-D treatment mimicked the protective effect of eBL. Next we tested whether EDTA could rescue young protocorms from the deleterious growth effects of 2,4-D in a similar manner. Six concentrations of EDTA (10μM – 10 mM) were tested, and while low levels provided no recovery, the higher concentration induced death. These results do not necessarily preclude a role for free radical-protection in the BR response of young seedlings to 2,4-D, but these findings suggest that BRs are likely providing other means to restore normal growth, such as mediating auxin flow.

### Regulation of Protocorm Hair Development by Brassinosteroids

In this work, eBL consistently repressed protocorm hair development, including those seedlings in which abundant hair formation had been induced by auxins. Work done with Arabidopsis has shown that BRs inhibit root hair formation through the up-regulation of GL2 and WER which represses RDH6 expression, a transcription factor needed for inducing hair cell fate (Cheng et al., 2014). Thus, similar gene regulation may be occurring in the orchid as well. However, PAT may also play a role in this inhibitory action by eBL. During root hair development, the future root hair cell accumulates auxin as a result of basipetal (shootward) flow via PIN2 proteins (Lee and Cho, 2013; Velasquez et al., 2016). The importance of this transport is evident in Arabidopsis *pin2* mutants which possess short root hairs (Rigas et al., 2013). In this study, the PAT inhibitors, TIBA, BFA, and monensin, caused seedlings to have a higher percentage of hair initiation (hair buds) over hair outgrowth when compared to the control seedlings. Since auxin signaling is required for root hair growth (Kim et al., 2006; Velasquez et al., 2016), the lack of auxin acquisition through PIN2 transport may be the reason for initiation over hair outgrowth that was evident in the PAT inhibitor-treated seedlings. Epibrassinolide application was unable to rescue this condition (data not shown), a response that may be a result of BR-induced expression of IAA/AUX genes that inhibit auxin signaling in root hair growth (Kim et al., 2006). Additional evidence supportive of the PAT- mediated hair outgrowth hypothesis, is the fact that BRZ- treated seedlings exhibited high initiation over hair outgrowth, suggesting that BR-enhanced auxin flow is needed for completion of protocorm hair formation.

The BRZ-induced, high hair initiation response was even more prominent in the auxin-treated seedlings, which typically produced high numbers of elongated protocorm hairs. BRZ caused nearly all hair initiation over hair outgrowth, regardless of the high auxin levels present. This finding is somewhat perplexing, since BRs have been shown to increase expression of IAA/AUX and inhibit auxin signaling (Kim et al., 2006), and it follows that a reduction in BRs would release this inhibition. The 2,4-D, IAA, and NAA-treated seedlings had plenty of auxin to promote hair cell growth, but, strangely, were unable to do this without BRs. Moreover, data from this study using BBo with BRZ demonstrated that auxin and BR biosynthesis are both needed for hair initiation/formation. Inhibition of auxin biosynthesis yielded more seedlings with hairs than the BRZ/BBo treatment. It is possible that BRs have a regulatory role in promoting protocorm hair development that is somehow balanced with its inhibitory effects.

Protocorm hair formation in *S. plicata* occurs in small clusters, resulting in many hair tufts on a given protocorm (Novak and Whitehouse, 2013). BRZ treatment of seedlings that had just begun hair initiation (30 DAC), yielded very prominent hair buds that were arranged in more numerous clusters and had more hair cells per cluster than in the control or eBL-treated seedlings. These findings are similar to the work in Arabidopsis where BRZ-treated Arabidopsis roots generated hairs from cells normally designated as non-hair cells (Cheng et al., 2014). Thus, anomalous hair patterning by lack of BR biosynthesis is evident in both systems, orchid and Arabidopsis, whose hairs differ in organ origin, protocorm and root, respectively. In contrast, the lack of BR biosynthesis resulted in a distinctive difference in the two systems, BRZ-treated Arabidopsis roots produced hairs of normal length (Cheng et al., 2014), while, in this study, BRZ consistently promoted hair initiation and inhibited hair outgrowth. Thus the requirement of BR biosynthesis for hair elongation may be unique to orchid protocorms.

## Conclusion

Unveiling mechanisms that regulate PAT is needed to better understand key morphological events in plant development, including shoot and hair formation. This study provided evidence to support BR-mediated PAT during protocorm and leaf development in *Spathoglottis*. Moreover, this work demonstrated that the role of BRs in hair formation may be more complex than previously thought; since results in this study also suggested that BRs may be involved in hair outgrowth and patterning on developing protocorms of orchids.

## Author Contributions

SN designed the experiments, conducted data analysis, and wrote the manuscript. NK, KU, and JdL contributed to the conception of the project, performed the experiments, and collected the data. WN collected data and conducted the SEM work. JJ assisted with the microscopy. All authors, reviewed, edited, and approved the final manuscript.

## Conflict of Interest Statement

The authors declare that the research was conducted in the absence of any commercial or financial relationships that could be construed as a potential conflict of interest.

## Supplementary Material

FIGURE S1 Growth parameters for data collection on a typical 25 DAC orchid seedling.
