# Supplementary figures and images for: Evidence for Brassinosteroid-Mediated PAT During Germination of Spathoglottis plicata (Orchidaceae)
Source: Front Plant Sci. 2018 Aug 17;9:1215. doi: 10.3389/fpls.2018.01215 (PMC6107755; doi:10.3389/fpls.2018.01215)

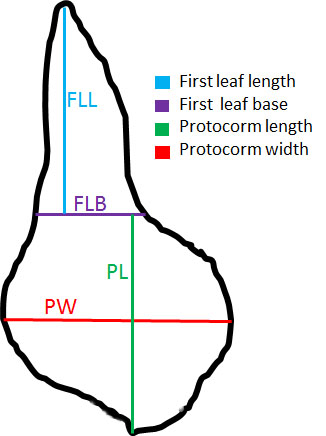

Supplement: FIGURE S1 [file Image_1.jpg]
